# Supplementary material for: Deletion of sulfate transporter SUL1 extends yeast replicative lifespan via reduced PKA signaling instead of decreased sulfate uptake
Source: eLife. 2025 Sep 3;13:RP94609. doi: 10.7554/eLife.94609 (PMC12408066; doi:10.7554/eLife.94609)
Supplement: Supplementary file 1. [file elife-94609-supp1.docx]

Supplementary File 1. Strains Used in this Study, Related to Experimental Procedures

| **Strain** | **Genotype** |
| --- | --- |
| BY4741 sul1Δ | MATa *his3Δ1 leu2Δ0 met15Δ0 ura3Δ0 sul1Δ::KanMX* |
| BY4741 sul2Δ | MATa *his3Δ1 leu2Δ0 met15Δ0 ura3Δ0 sul2Δ::KanMX* |
| BY4741 met3Δ | MATa *his3Δ1 leu2Δ0 met15Δ0 ura3Δ0 met3Δ::KanMX* |
| BY4741 msn2Δ | MATa *his3Δ1 leu2Δ0 met15Δ0 ura3Δ0 msn2Δ::his* |
| BY4741 atg8Δ | MATa *his3Δ1 leu2Δ0 met15Δ0 ura3Δ0 atg8Δ::his* |
| BY4741 sul1Δmsn2Δ | MATa *his3Δ1 leu2Δ0 met15Δ0 ura3Δ0 sul1Δ::KanMX msn2Δ::his* |
| BY4741 sul1Δatg8Δ | MATa *his3Δ1 leu2Δ0 met15Δ0 ura3Δ0 sul1Δ::KanMX atg8Δ::his* |
