# Supplementary material for: Deletion of sulfate transporter SUL1 extends yeast replicative lifespan via reduced PKA signaling instead of decreased sulfate uptake
Source: eLife. 2025 Sep 3;13:RP94609. doi: 10.7554/eLife.94609 (PMC12408066; doi:10.7554/eLife.94609)
Supplement: Supplementary file 2. [file elife-94609-supp2.docx]

Supplementary File 2. Primers Used in this Study, Related to Experimental Procedures

| **Primer** | **Sequence** |
| --- | --- |
| ko-SUL1-F | GAATATGTCACGTAAGAGCTCGACTGAATATGTGCATAATCAGGAGGATGgacatggaggcccagaat |
| ko-SUL1-R | CGGGTATATCGATATGAAAAAACGGTAAATTTGTCCCAGTTGCAGCACATcagtatagcgaccagcat |
| ko-SUL2-F | GCAGGGAATTATCTAAGATATGTCCAGGGAAGGTTATCCAAACTTTGAAGgacatggaggcccagaat |
| ko-SUL2-R | GGGATATCGATATGGAAGAAAGGTAGATTTGTTCCTGAAGCGGTATAAACcagtatagcgaccagcat |
| ko-MET3-F | CATGCCTGCTCCTCACGGTGGTATTCTACAAGACTTGATTGCTAGAGATGgacatggaggcccagaat |
| ko-MET3-R | GGTCGATCATGAATTTTGCCCTACTTTTGAGATGGGAGCATTTTATGACGcagtatagcgaccagcat |
| ko-ATG8-F | ATGAAGTCTACATTTAAGTCTGAATATCCATTTGAAAAAAGgacatggaggcccagaat |
| ko-ATG8-R | CTACCTGCCAAATGTATTTTCTCCTGAGTAAGTGACATACAcagtatagcgaccagcat |
| ko-MSN2-F | CAACTTTTATTGCTCATAGAAGAACTAGATCTAAAATGACGGTCGACCATgacatggaggcccagaat |
| ko-MSN2-R | GGGGTCTATTAAATGTCTCCATGTTTTTTATGAGTCTTGATGTGTTGCGAcagtatagcgaccagcat |
| ko-MSN4-F | TAATGCTAGTCTTCGGACCTAATAGTAGTTTCGTTCGTCACGCAAACAAGgacatggaggcccagaat |
| ko-MSN4-R | ACTTGTCATACCGTAGCTTGTCTTGCTTTTATTTGCTTTTGACCTTATTTcagtatagcgaccagcat |
| MSN2-EGFR-F | GCGATAATTTGTCGCAACACATCAAGACTCATAAAAAACATGGAGACATTggtggttctggtggtggttct |
| MSN2-EGFR-R | CAATAAGCCGTAAGCTTCATAAGTCATTGAACAGAATTATCTTATGAAGcagtatagcgaccagcattc |
| MSN4-EGFR-F | GTGACAATTTATCACAACATCTAAAAACTCACAAAAAGCACGGTGATTTTggtggttctggtggtggttct |
| MSN4-EGFR-R | CTGAGGAAGAAAGAATATTATTTCTCCGAAAACTTGTCATACCGTAGCTTcagtatagcgaccagcattc |
| ATG8-EGFR-F | AGGACGGGTTTTTGTATGTCACTTACTCAGGAGAAAATACATTTGGCAGGggtggttctggtggtggttct |
| ATG8-EGFR-R | CATTCTTATACTGGAACAATAGATGGCTAATGAGTCCCTATAATTTCGAcagtatagcgaccagcattc |
| E427Q-mutantF1 | GACGGCCAGTGAATTCATGTCACGTAAGAGCTCGACTGAATATGTGCATAAT |
| E427Q-mutantR1 | ACTACACTACTTGTCATCGTCGTCCTTGTAATCAACGTCCCATTTAGAAAAATCGGGTATATCGATA |
| E427Q-mutantF2 | TGACAAGTAGTGTAGTAGATAGTAAGTACTTTAATTACCCCCCCTGT |
| E427Q-mutantR2 | TATTCTGGGCCTCCATGTCCTCGAGCACATTAGGAGAGACAAGCCGTCCAAACT |
| E427Q-mutantF3 | GTCTCTCCTAATGTGCTCGAGGACATGGAGGCCCAGAATACCCTCCTTGACAG |
| E427Q-mutantR3 | GATTACGCCAAGCTTCAGTATAGCGACCAGCATTCACATACGATTGA |
| E427Q-mutantF427 | GTTGTCCCTGACCAACAACTTATTGCGATTG |
| E427Q-mutantR427 | CAATCGCAATAAGTTGTTGGTCAGGGACAAC |
| q-SUL1-F-1 | TGAGGTTTTACCAGCCCCAG |
| q-SUL1-R-1 | TGCCTTGAGCAGTTCACGTA |
| q-SUL2-F-1 | CCGACCAAGAATTGATTGCT |
| q-SUL2-R-1 | AAGAATGCGCCGGTCAAACA |
| q-MET3-F | GGTCCATACGATGCTCAAGA |
| q-MET3-R | CTTGTTTTGGTCTTGGTGGG |
| q-MSN2-F | CCAAGTCAGCAATTACAGCA |
| q-MSN2-R | CGGCAGCATATCGTTCTTGG |
| q-MSN4-F | TCGCGACGCAAGAAGATACA |
| q-MSN4-R | GTTGATCCACGCCTCTCAGT |
| q-ATG8-F | AGTTCCTGCTGACCTTACCG |
| q-ATG8-R | ACCCGTCCTTATCCTTGTGTT |
| q-HSP12-F | ACATCACTGACAAGGCCGAC |
| q-HSP12-R | GCGGCTCCCATGTAATCTCT |
| q-ALD3-F | ACATCTTCTGAGCAACGTGGTA |
| q-ALD3-R | CCGCCCCCGCATAGTATCTT |
| q-RTN2-F | GTTCACGAGTTTTGCGGTGG |
| q-RTN2-R | TTCTGTCCTCTAACGCAGGC |
| q-SIP18-F | GGAAAGAACGCCAAATCCTCC |
| q-SIP18-R | TCCAATCGTTCGCAATTCCTC |
| q-CTT1-F | GCAATTCCACGTCTTGTCGG |
| q-CTT1-R | AGTTGCTTGTTCGGGTGTCA |
| q-TPS1-F | GCCGTACCCATCTTCCTGAG |
| q-TPS1-R | GGTTTGCCTCGTTGTATGCC |
| q-PNS1-F | TATGCACGGTAGGCGGATTC |
| q-PNS1-R | GGATGGCAGCATTGGTGTTC |
| q-ACT1-F-1 | CGTTTCCATCCAAGCCGTTT |
| q-ACT1-R-1 | ACCGGCCAAATCGATTCTCAA |
